# Modelling of a double‐scattering proton therapy nozzle using the FLUKA Monte Carlo code and analysis of linear energy transfer in patients treated for prostate cancer

**DOI:** 10.1002/acm2.70032

**Published:** 2025-03-19

**Authors:** Rasmus Klitgaard, Lars Fredrik Fjæra, Camilla Hanquist Stokkevåg, Perry Johnson, Mark Artz, Nancy Price Mendenhall, Curtis Bryant, Ludvig Paul Muren

**Affiliations:** ^1^ Danish Centre for Particle Therapy Aarhus University Aarhus N Denmark; ^2^ Department of Medical Physics Oslo University Hospital Oslo Norway; ^3^ Department of Physics and Technology University of Bergen Bergen Norway; ^4^ Department of Radiation Oncology College of Medicine University of Florida Health Jacksonville Florida USA

**Keywords:** FLUKA, linear energy transfer, Monte Carlo, prostate cancer, proton therapy

## Abstract

**Background:**

The dose‐averaged linear energy transfer (LET_D_) in proton therapy (PT) has in pre‐clinical studies been linked to the relative biological effectiveness (RBE) of protons. Until recently, the most common PT delivery method in prostate cancer has been double‐scattered PT, with LET_D_ only available through dedicated Monte Carlo (MC) simulations. However, as most studies of the relationship between LET_D_ and RBE in double scattered PT have been focused on the head and neck region, existing MC implementations have not been capable of calculating LET_D_ for the longer field ranges used, for example, in the pelvic region.

**Purpose:**

The initial aim of this study was to implement a MC code allowing for LET_D_ calculations in double‐scattered PT of prostate cancer. Additionally, we explored LET_D_ profiles and LET_D_ as a function of field configuration, by performing MC calculations for a large prostate cancer cohort treated with double‐scattered PT.

**Methods:**

The components of a passive scattered clinical treatment nozzle used for delivery of extended field ranges, with two associated modulation wheels, were implemented into an existing FLUKA MC framework for LET_D_ calculations. The code was validated to spread out Bragg peak (SOBP) measurements conducted using the treatment nozzle with 11 different range and modulation width configurations. After validation, LET_D_ distributions were calculated on the planning computed tomographies of 582 prostate cancer patients treated with two‐field double‐scattered PT. All patients had symmetric field configurations with respect to the sagittal plane, with one pair of posterior oblique, lateral opposing, or anterior oblique fields. Dose and LET_D_ volume parameters and the mean LET_D_ ratio between the bladder and rectum were compared across the three groups.

**Results:**

The range differences were below 1 mm for all SOBP scenarios used for calibration. For 9 of 11 SOBP scenarios, the modulation width differences were below 2 mm. For the patient simulations, the mean gamma pass rates (3 mm/3%) were at least 98% in the PTV, bladder, and rectum. Comparing anterior to posterior field configurations, the mean LET_D_ in the bladder increased within both the 10 and 70 Gy iso‐dose regions, and conversely, the mean LET_D_ decreased for the rectum. There was a marked difference in the mean bladder‐to‐rectum LET_D_ ratios between anterior oblique, lateral opposing and posterior oblique field configurations.

**Conclusion:**

A MC code allowing for accurate calculations of dose and LET_D_ in double‐scattered PT of prostate cancer was implemented and validated. The LET_D_ distributions in the rectum and bladder showed a systematic dependence on the field configuration.

## 1 INTRODUCTION

Proton therapy (PT) is potentially a more conformal treatment modality for cancer than photon‐based radiotherapy (RT), due to the characteristic charged particle interactions with matter resulting in a favorable depth dose curve. PT is therefore currently used for several tumor sites, including the pelvis in general and prostate in particular.^1^, ^2^, ^3^, ^4^, ^5^, ^6^ Although most new PT installations are applying pencil beam scanning (PBS) beam delivery, historically, most prostate cancer patients having received PT have been treated with double‐scattered PT. Further, the majority of PT follow‐up data was collected on patients treated with double‐scattered PT, and currently, 37% of PT centres still employ passive scattering.^7^ The normal tissue sparing potential of both modalities of PT depends not only on the improved treatment conformity, but also on a thorough knowledge about the relative biological effectiveness (RBE) of protons compared to photons. PT has a higher effectiveness compared to photon‐based RT, with a current recommended value for the RBE of 1.1.^8^ However, in vitro cell studies have shown that the RBE varies with both dose, fractionation, tissue type, and dose‐averaged linear energy transfer (LET_D_).^9^ The dependencies of RBE to these factors, including LET_D_, are currently receiving considerable attention.^10^, ^11^, ^12^, ^13^, ^14^

LET_D_ expresses the physical energy deposition per unit path length, averaged over the dose deposited by the particle. In a proton field, LET_D_ increases toward the end of the depth‐dose curve, enhancing the RBE beyond the distal end of a treatment field. The three‐dimensional LET_D_ distribution can be determined using Monte Carlo (MC) simulations, which is considered the gold standard calculation method. MC simulations of double scattering PT delivery systems are more time‐ and resource‐intensive compared to PBS implementations since phase‐space approaches are not applicable in MC simulations of double‐scattered PT.^15^ Further, most MC studies of LET_D_ calculations for clinical double‐scattered PT have focused on brain and other tumor sites with shorter ranged treatment fields and have therefore not implemented geometry used in calculations of longer ranged treatment fields, such as those used in PT of prostate cancer.

MC simulations of double‐scattered PT are usually not applied in clinical practice due to the heavy computational load,^16^ however, the potential effects of LET_D_ can be considered by carefully selecting beam angles and the locations of distal edges.^17^ In PBS PT of prostate cancer, the high LET_D_ region has been shown to not only be placed in the distal end of the treatment field, but also at the lateral penumbra of the spread out Bragg Peak (SOBP).^18^ Whereas adjustment of the beam configuration in treatment of prostate cancer was applied to modulate the physical dose distribution, the implications for the associated LET_D_ distributions is uncertain.

The aim of this study was therefore to initially implement the necessary geometry for re‐calculations of treatment plans for prostate cancer, with verification of dose calculations to measurements, and subsequently to analyze the field‐angle dependence on high‐dose LET_D_ in both the rectum and bladder in a large patient cohort.

## 2 MATERIALS AND METHODS

### 2.1 The double scattering treatment nozzle

The operation of the double scattering PT delivery system investigated (IBA Universal nozzle, University of Florida Health Proton Therapy Institute (UFHP) was characterized by the three main characteristics: the delivered dose, the spatial profile of the proton beam, and the energy distribution of the beam.^15^, ^19^, ^21^ The delivered dose was dependent on the monitor units delivered along with the output factor of the treatment field. The spatial profile was described by the field radius and the lateral shape of the beam. Three main components conformed the beam to the required shape: (i) the beam was broadened by the second scattering shield, (ii) narrowed by the variable collimators and the snout, and (iii) conformed to the specific target shape by the treatment field specific aperture. The energy modulation of the beam was performed primarily by two components, (i) the rotation of the range modulation wheel, which broadened the energy distribution to match the required SOBP, and (ii) the treatment field specific range compensator which conformed the energy distribution to match the distal shape of the target.

**FIGURE 1:**
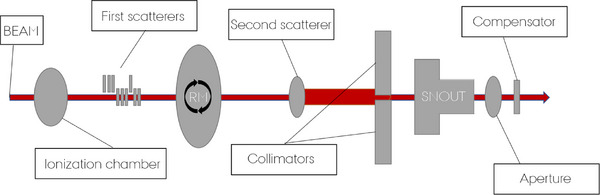
Sketch of the double scattering proton therapy treatment nozzle. Distances and sizes are not to scale. The second ionization chamber and the field mirror are not visualised. The proton beam is traversing from left to right and shown as the red line.

Of the mentioned components, two long‐range specific range modulation wheels needed implementation and following dose validation in this study. A more detailed description of the FLUKA MC implementation was published by Fjæra et al. In 2020^15^ (see also Figure 1).

### 2.2 Implementation and validation of range modulation wheels

Two long‐range specific range‐modulation wheels were used under treatments of prostate cancer and required implementation into the FLUKA MC code. The range‐modulation wheels were implemented according to manufacturer blueprints, and each consisted of three plates: An upstream plate made from a low‐Z material (Lexan and Carbon), a center plate made from aluminium, and a downstream plate made of lead. The aluminium enter plates had fixed thicknesses of 1 mm, while the up‐ and downstream plates had steps of increasing and decreasing thicknesses around the circumference of the plate, respectively. To ensure no overlap of the largest and smallest range pull‐back, the upstream plate had a brass block between the thickest and thinnest step. All components were implemented in FLUKA using the Flair geoviewer^22^ as cylinders with each step confined to its respective angular segment. The zero‐angle of the range modulation wheels was not supplied in the blueprints and had to be estimated through matching dose‐depth simulations with dose‐depth experimental measurements.

The SOBP modulation width of each field delivery was translated into a rotation angle of the range modulation wheel, starting at the zero‐angle. To ensure a consistent energy modulation, the zero angle of the beam was within the angular segment of the brass block when the beam was initially turned on. After the beam was turned on, the range modulation wheels were rotated an angle equivalent to the rotation angle, defined from the requested modulation width. After this rotation, the beam was turned off and returned to the zero‐angle. This was repeated several times per SOBP and combined with beam current modulation resulted in a flat SOBP. The final position of the proton beam on the range modulation wheel, and thus also the modulation‐width of the delivered SOBP, was therefore dependent on the requested SOBP as well as the zero‐angle. Therefore, by comparing measured SOBPs with simulated SOBPs, it could be deduced whether the MC simulation employed a zero‐angle too small or too large, and subsequently, the zero‐angle could be iterated.

The range was defined as the distance to the distal D80% and the modulation width was defined as the range from the proximal D90% to the distal D90%. The accuracy goals were 1 mm for range differences and 2 mm for modulation width differences.

The measurements were obtained as described by Fjæra et al.^15^ and employed a square aperture with an opening of 15 cm × 15 cm and an air gap of 10 cm. The dose was measured with an IBA PPC05 parallel plate ionization chamber with 1 cm collection diameter, placed in an IBA Blue Phantom water phantom and irradiated with a gantry angle of 0 degrees to avoid the phantom wall. We did not conduct additional measurements in a phantom representative of the pelvic anatomy as the treatment nozzle was decommissioned prior to this study. The depth wise measurements were conducted every 0.5 mm. Five measurements were conducted for one range modulation wheel and six for the other, for 11 total SOBP irradiations over the two range modulation wheels.

FLUKA MC calculations with the same setup as the experimental measurements were performed with dose scored in a cylinder with a 2 cm diameter and a depth wise resolution of 0.5 mm along the path of the proton beam. The dose was normalised to the mean dose in the centre of the SOBP ±25% of the requested modulation width. An iterative search through zero‐angles was employed, starting with an angle of 0 degrees, using 10 million primaries per simulation. As the differences in modulation‐widths between the calculations and the measurements reached a minimum, a grid search of +‐3.5 degrees in the vicinity of the minimum was initiated, with 0.5 degree spacing, and 100 million primaries per simulation. All simulations were conducted with FLUKA2021.2.x, using the default HADROTHE settings.

### 2.3 Field angle dependence on LET

Dose and LET_D_ distributions were calculated for 582 prostate cancer patients using the FLUKA MC implementation. All patients were treated using the implemented double scattering PT nozzle at UFHPTI in the years 2006–2010 in an ethical board approved protocol with lateral opposing fields, symmetric anterior oblique fields, or symmetric posterior oblique fields.

The clinical target volumes (CTVs) included the prostate for low, intermediate, and high‐risk patients, while also the proximal 2 cm of the seminal vesicles were included in the CTV for the intermediate and high‐risk patients. The patients were planned using a planning target volume (PTV) with an 8 mm margin in the superior‐inferior directions and a 6 mm margin in axial directions (lowered to 6 and 4 mm, respectively, after 2008).

For each patient, the clinical treatment plan was recalculated using FLUKA MC, scoring dose, and LET_D_ in water for all protons in the planning dose distribution grid, with MC dose normalised to the median planning dose in the CTV. To ensure accuracy, the MC dose was compared to the planning dose using gamma pass analysis with 3 mm/3% and 2 mm/2% criteria for all voxels over 10% of the global max dose. The MC simulations were computed on a highly parallelised distributed computing system with 200 million primaries per field to ensure a low statistical uncertainty (1 standard deviation). The computation times were roughly 1.5 h per patient.

The mean LET_D_ values in the rectum and bladder were compared in the 10 and 70 Gy iso‐dose regions across all patients grouped by treatment field configuration: anterior oblique, lateral opposing, and posterior oblique. In total, there were nine unique field angle configurations over all patients (*n* = 582), with five different configurations in the anterior oblique group (*n* = 471), one in the lateral opposing (*n* = 37), and three in the posterior oblique group (*n* = 74). All angles were within ± 10 degrees of lateral opposing. Moreover, to investigate the impact of potential LET_D_ optimization through altering field angles, the mean LET_D_, $\bar{{\mathrm{LET}}_{D}}\left(D_{T}\right)$, for all voxels of the bladder and rectum receiving more than a threshold dose, D_T, was calculated for D_T ranging from 0 to 80 Gy in steps of 0.1 Gy. The ratios of $\bar{{\mathrm{LET}}_{D}}\left(D_{T}\right)$ in the bladder and rectum between patients with anterior oblique, direct lateral opposing, and posterior oblique field configurations were compared. The bladder‐to‐rectum ratios of $\bar{{\mathrm{LET}}_{D}}\left(D_{T}\right)$ were compared using 95% confidence intervals, with qq‐plots being used to check for normality at each dose level.

## 3 RESULTS

### 3.1 Implementation and validation of range modulation wheels

**FIGURE 2:**
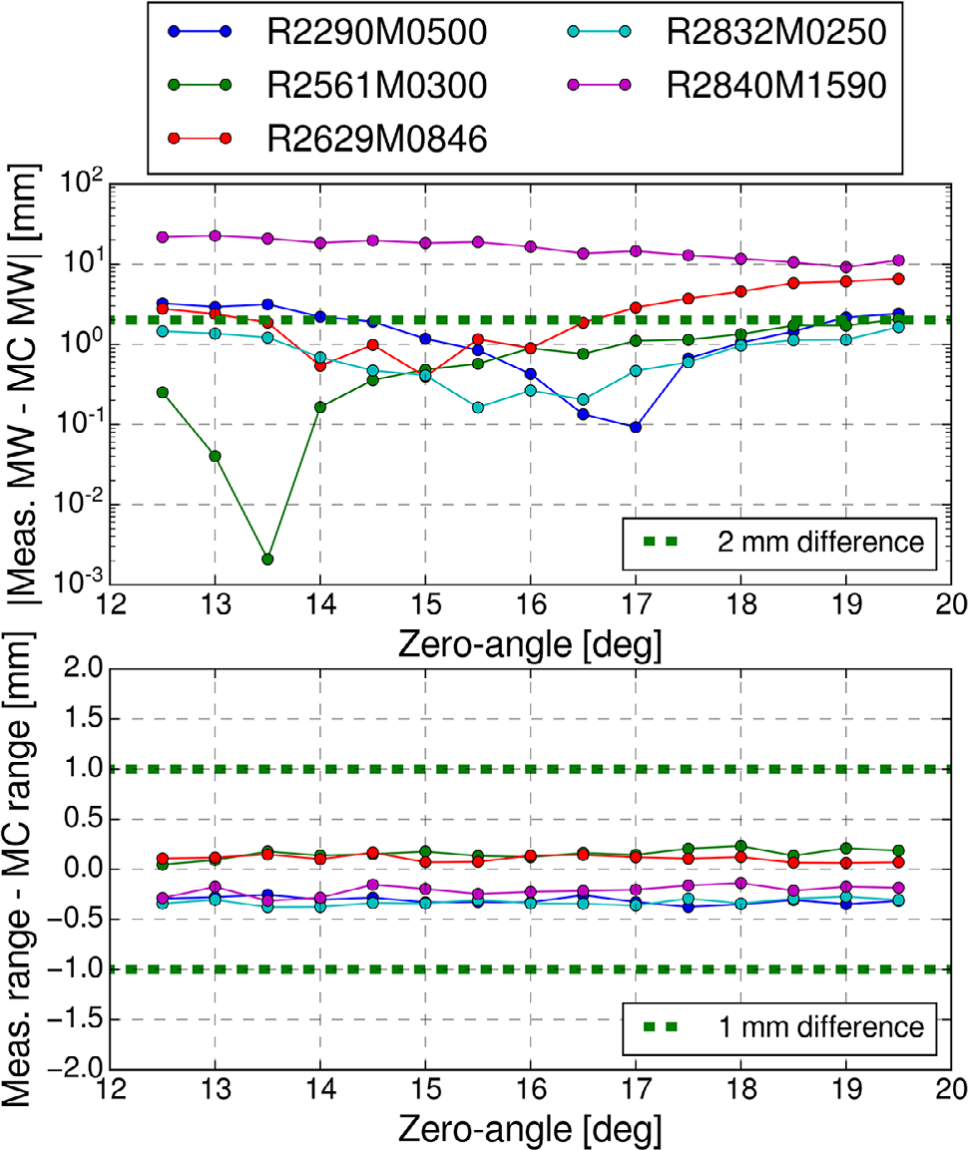
Top‐panel: Absolute differences in modulation widths (MW) between FLUKA MC simulations and measurements as a function of MC simulation zero‐angle for range‐modulation wheel 1. Note the dashed green line indicating a difference of 2 mm. Bottom panel: Range differences between FLUKA MC simulations and measurements as a function of MC simulation zero‐angle for range‐modulation wheel 1. The difference is calculated as the FLUKA MC range subtracted from the measured range and the dashed green lines indicate plus and minus 1 mm range. The requested ranges and modulation widths are written as RxxxxMyyyy, where xxxx is the range in 10^−1^ mm and yyyy is the modulation width in 10^−1^ mm.

**FIGURE 3:**
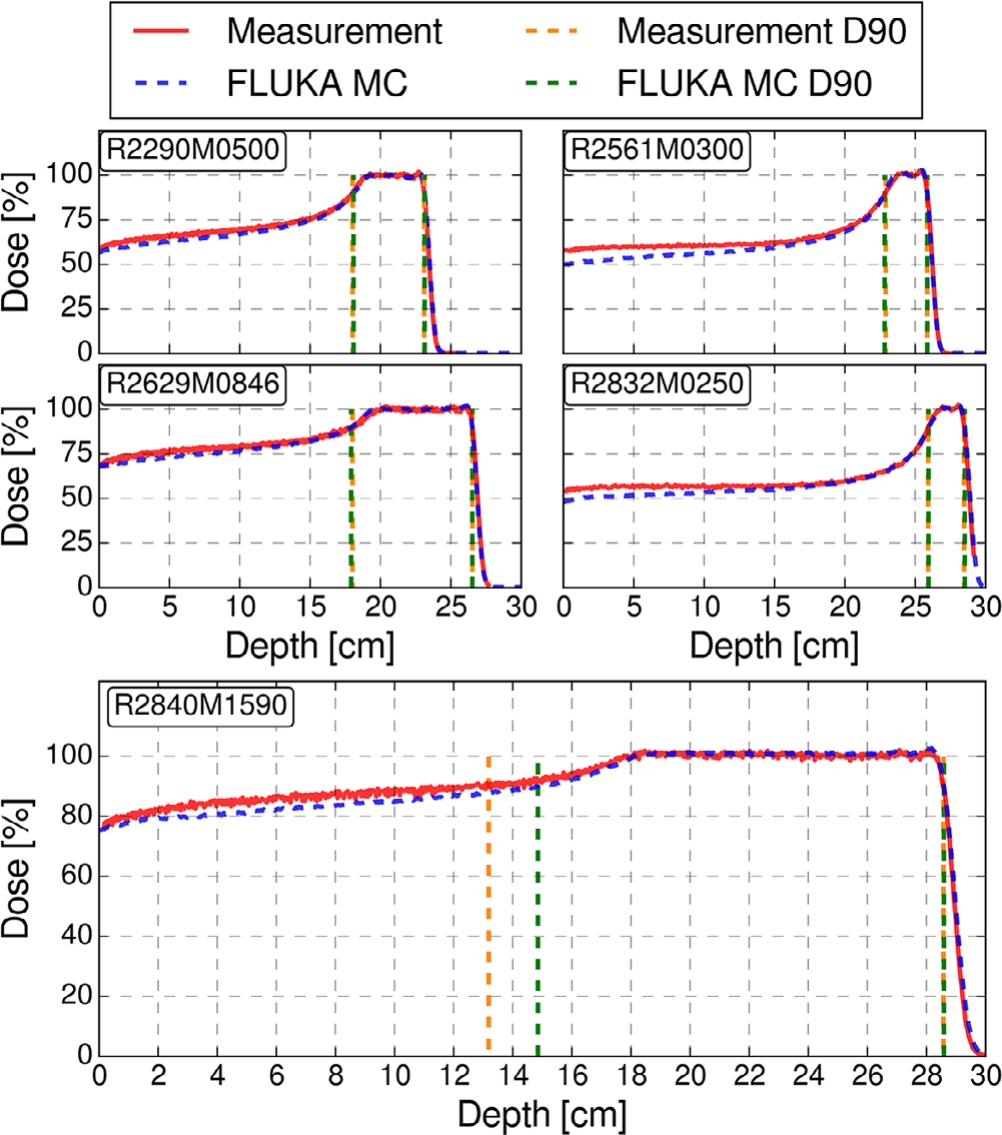
Dose depth curves for all measured and simulated SOBPs using range modulation wheel 1. The dashed blue lines represent the FLUKA MC calculated dose, and the red line represents measurements. The dashed vertical lines represent the D90% ranges (proximal and distal) for FLUKA MC (green) and measurements (orange). The requested ranges and modulation widths are written as RxxxxMyyyy in the upper left corners, where xxxx is the range in 10^−1^ mm and yyyy is the modulation width in 10^−1^ mm.

The best fitting zero‐angles were identified in the iterative search, resulting in SOBP ranges within 1 mm of measurements for all SOBPs, and ten of eleven simulated SOBP modulation widths within 2 mm of measurements, see Figure 2 for the zero‐angle dependence of range modulation wheel 1. The SOBP with the largest difference in modulation width had a deviation of 16.5 mm, while all simulated SOBPs were visually close to measurements apart from the proximal dose of long‐range fields, as seen in Figure 3 for range‐modulation wheels 1 (see Supplementary Figure B for range‐modulation wheel 2). The best fitting zero‐angles were chosen as 16.0  and 28.0 deg for range‐modulation wheels 1 and 2, respectively. A full overview of the ranges and modulation widths, as well as the differences between simulations and measurements can be found in Table 1 (validation data for range‐modulation wheel 2 can be found in Supplementary Figures A and B).

**TABLE 1:** Overview of all measured and simulated SOBPs with their requested ranges and modulation widths along with the differences between measurements and FLUKA MC calculations, calculated as the measured values subtracted from the FLUKA MC results.

Modulation wheel	Requested range (cm)	Requested modulation width (cm)	Difference range (mm) FLUKA ‐ Measured	Difference modulation width (mm) FLUKA ‐ Measured
1	19.84	3.00	0.0	−0.7
	20.74	11.50	−0.5	−0.9
	21.87	15.90	−0.0	−1.9
	22.10	6.00	0.5	0.9
	23.46	11.00	0.4	0.2
	23.91	16.00	−0.2	1.3
2	22.90	5.00	−0.3	−0.4
	25.61	3.00	0.1	0.9
	26.29	8.46	0.1	0.9
	28.32	2.50	−0.3	0.3
	28.40	15.90	−0.2	−16.5

### 3.2 Field angle dependence on LET_D_

The mean gamma pass rates of the patient recalculations were 95% in the body and above 98% in PTV, bladder, and rectum with a 3 mm/3% requirement and 89% in the body and above 94% in PTV, bladder, and rectum using 2 mm/2% requirement. All simulations achieved mean statistical uncertainties below 1% in the CTV.

**FIGURE 4:**
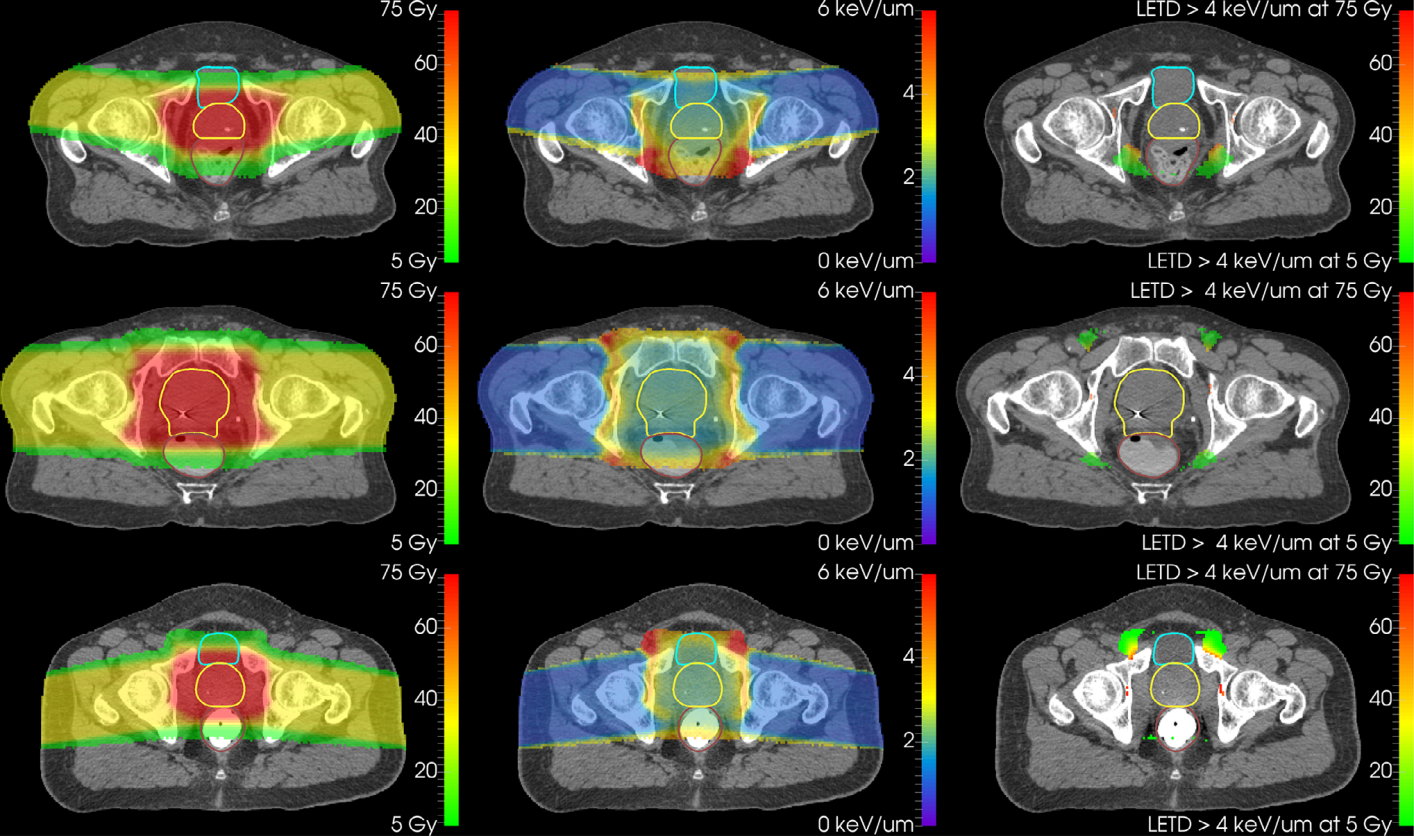
Dose, LET_D_, and dose with LET_D_ > 4 keV/um distributions for three patients, shown in the central axial plane of the prostate. Contours are, from the bottom to the top of each picture, rectum, CTV, and bladder (if present). Only voxels receiving more than 2 Gy are colourised. Top row: Patient treated with anterior oblique fields (82/278). Middle row: Patient treated with lateral opposing beams (90/270). Bottom row: Patient treated with posterior oblique fields (98/262).

**FIGURE 5:**
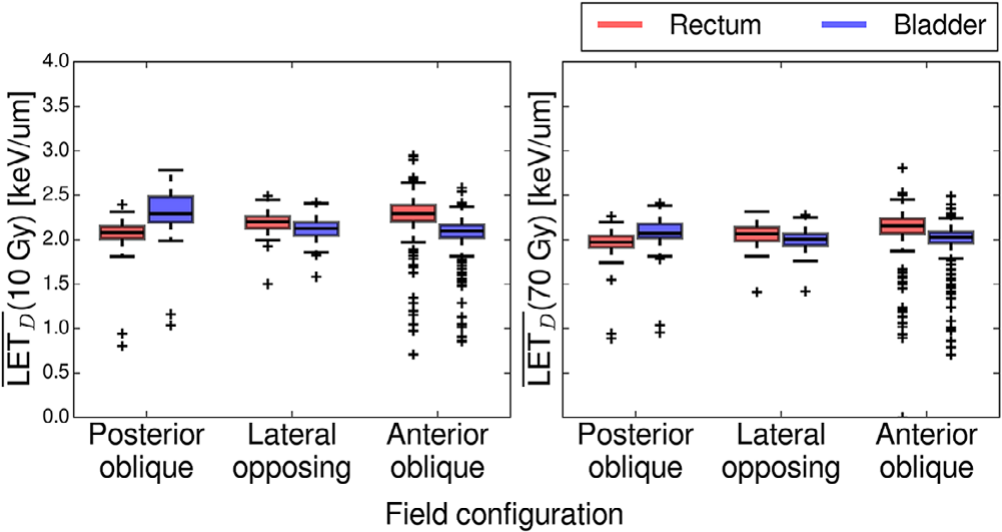
Boxplots of mean LET_D_ above two dose thresholds in the rectum (orange) and bladder (blue) for patients treated with symmetric anterior oblique, lateral opposing or symmetric posterior oblique fields. The dose thresholds used were 10 and 70 Gy in the left and right panel, respectively. The posterior oblique group accounts for 74 patients, while the lateral opposing and anterior oblique groups contain 37 and 471 patients, respectively.

Higher values of LET_D_ were located in the anterior region of the pelvis for posterior oblique fields, and vice versa for the anterior oblique fields as seen in Figure 4. Very high LET_D_ (> 4 keV/um) regions reached into the rectum for anterior oblique fields and slightly into the bladder for posterior oblique fields (see Figure 4). The mean LET_D_ values in the bladder were generally larger than the mean LET_D_ values in the rectum for the patients treated with posterior oblique fields, as seen in Figure 5, with median‐differences (calculated as rectum—bladder) in the 10 Gy iso‐dose of ‐0.22 , 0.08 , and 0.19 keV/um for the posterior oblique, lateral opposing, and the anterior oblique groups, respectively. In the 70 Gy iso‐dose, the same trend was seen; however, the differences were less pronounced, with median‐differences of ‐0.11 , 0.06, and 0.13 keV/um for the same three groups.

**FIGURE 6:**
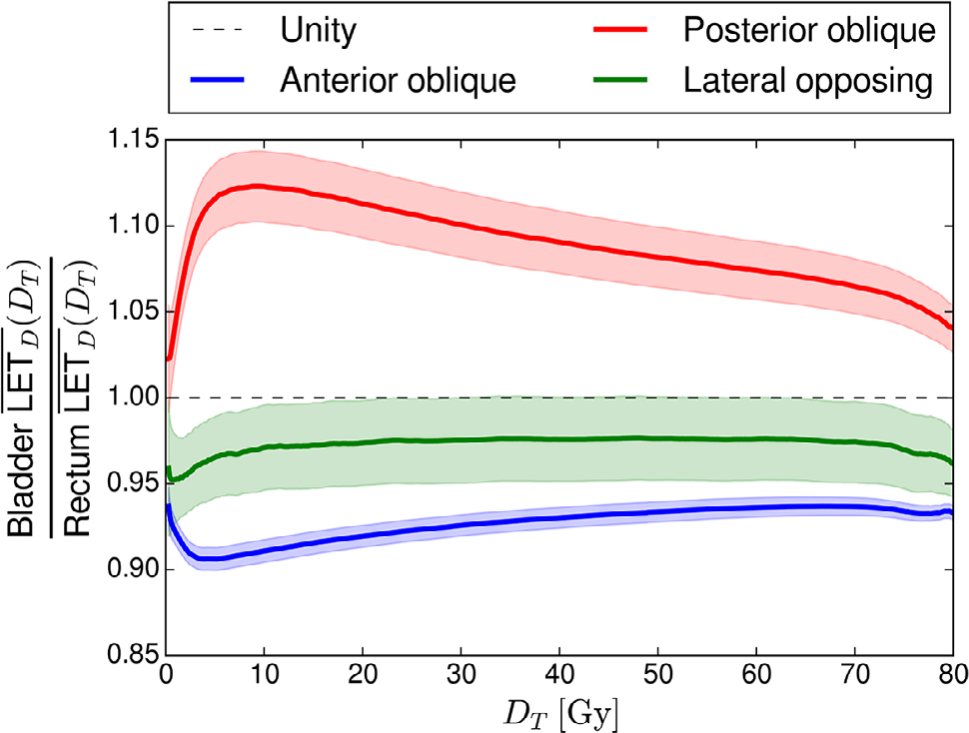
Ratio of $\bar{{\mathrm{LET}}_{D}}\left(D_{T}\right)$ between bladder and rectum for all patients, grouped into anterior oblique, lateral opposing, and posterior oblique fields scattering (blue, green, and red, respectively). The shaded regions represent the 95% confidence interval in each patient group and the solid lines represent the mean. The black, dashed line represents a ratio of 1, indicating an equal ratio. There are 471 patients in the anterior oblique group, 74 in the posterior oblique group, and 37 in the lateral opposing group.

As shown in Figure 6, the ratio of $\bar{{\mathrm{LET}}_{D}}\left(D_{T}\right)$ between the bladder and rectum was largest in the posterior oblique group for all dose thresholds, with a maximum mean value of 1.13 at a dose threshold of 9.6 Gy, while the anterior oblique group had the lowest ratio for all dose thresholds, with a minimum mean value of 0.91 at a dose threshold of 5.2 Gy. The $\bar{{\mathrm{LET}}_{D}}\left(D_{T}\right)$ in the posterior oblique group was highest in the bladder for all dose thresholds, while it was highest in the rectum for all dose thresholds in the anterior oblique group. The lateral opposing group had ratios in‐between the anterior oblique and posterior oblique groups for all dose thresholds and showed higher $\bar{{\mathrm{LET}}_{D}}\left(D_{T}\right)$ in the rectum for all dose thresholds. The ratio of $\bar{{\mathrm{LET}}_{D}}\left(D_{T}\right)$ between the bladder and rectum was monotonously decreasing past the maximum for the posterior oblique group with the opposite effect observed in the anterior oblique group. The lateral opposing group displayed a flat profile of the ratio of $\bar{{\mathrm{LET}}_{D}}\left(D_{T}\right)$ between the bladder and rectum.

## 4 DISCUSSION

In this study, we implemented and validated the long‐range components in a double scattering PT nozzle using FLUKA MC. The results showed good agreement with experimental measurements. Subsequently, the implementation was used to calculate dose and LET_D_ in 582 prostate cancer patients with symmetric near‐opposing field configurations wherein the LET_D_ distributions for the bladder and the rectum was examined. Clear differences in bladder and rectum LET_D_ across anterior oblique, lateral opposing, and posterior oblique treatment fields were found.

All MC calculated ranges were within 1 mm of experimental measurements, and the modulation widths were within 2 mm for 10 of 11 calibration SOBPs. The calibration SOPB with a modulation width difference larger than 2 mm was for a treatment field with a modulation width of 15.9 cm and a range of 28.4 cm, and while the modulation width difference was 16.5 mm, visual inspection of the SOBPs (Figure 3 and Supplementary Figure B) revealed that the discrepancy was in the proximal dose region. These results were similar to what has been found in other MC implementations of double scattering treatment nozzles, and the cause is likely attributed to the definition of the modulation width.^15^, ^19^, ^23^, ^24^, ^25^ Fjæra et al. implemented the short‐range components of the same treatment nozzle studied here, where similar results were seen for large modulation widths.^15^ The modulation width was defined as the distance from the distal D90% to the proximal D90% which for fields with large modulation widths also included parts of the proximal build‐up region. To more accurately describe only the flat part of the SOBP, the modulation width could have been defined as the distance from the proximal D98% to the distal D90%, limiting the defined area to the flat parts of the SOBP.^26^

Differences in median LET_D_ values of the rectum and bladder within the 10 Gy iso‐dose varied between ‐0.22  and 0.19 keV/um across the three field angle configurations. These differences are generally small and a change in LET of this magnitude would account for a difference of roughly 0.01 in RBE when using the Unkelbach RBE model^27^ with *c* = 0.04. This difference is very small, and the clinical significance is likely minimal—it should be noted these are the median differences and local LET_D_ differences could be larger. There was a systematic difference between the anterior oblique and posterior oblique treatment fields, and while our study does not investigate individual field angles, we do expect this difference to be increasing with further treatment field angulation.

Furthermore, we calculated the mean LET_D_ ratio between the bladder and rectum across all dose thresholds for all 582 patients and found no overlap of the 95% confidence intervals between the three field‐angle groups. A result similar to this was also shown by Pedersen et al. in 2017^18^ for six patients with PBS PT. They found increasing LET_D_ in the rectum and bladder for anterior oblique and posterior oblique fields, respectively, but they primarily looked at near‐max LET_D_ values. We expect these differences in bladder to rectum ratios of $\bar{{\mathrm{LET}}_{D}}\left(D_{T}\right)$ to generalize across individual field angles; however, we were not able to show this using our dataset as some field angles had only been used in few cases.

In this study, we report the mean LET_D_ of volumes receiving more than D_T Gy as a function of the field configurations observed in this patient cohort. While other studies into LET_D_ and field angles exist,^18^, ^28^ they have primarily been planning studies based on few patients, so while our cohort is skewed toward anterior oblique fields, this analysis provides an overview of actual dose and LET_D_ distributions in a large prostate cancer cohort treated using double‐scattered PT with symmetric near‐opposing fields.

The patients included in the present study is part of a larger cohort of 1151 patients with prospectively registered morbidity data. The work in this study enables LET_D_ calculations for the entire cohort and are currently being used to develop an LET_D_‐inclusive statistical morbidity analysis. This could provide important information about the potential role of LET_D_ in morbidity models for prostate cancer specifically and for PT in general.

## 5 CONCLUSION

MC code required to perform dose and LET_D_ calculations on a double‐scattered PT prostate cancer cohort was implemented and validated. An overall good accuracy was achieved when comparing to measurements, thus allowing for dose and LET_D_ calculations in patients. Dose and LET_D_ calculations were performed in 582 prostate cancer cases and showed a systematic difference in the mean LET_D_ values of the bladder and rectum as well as the bladder‐to‐rectum ratio of mean LET_D_ between anterior oblique, lateral opposing, and posterior oblique treatment configurations.

## AUTHOR CONTRIBUTIONS

Rasmus Klitgaard: Wrote the manuscript, study design, data analysis, MC simulations development, data interpretation. Approves the final version of the manuscript. Lars Fredrik Fjæra: Study design, Monte Carlo simulations development, data interpretation. Approves the final version of the manuscript. Camilla Hanquist Stokkevåg: Study design, data interpretation, revising manuscript. Approves the final version of the manuscript. Perry Johnson, Mark Artz, Nancy Price Mendenhall, Curtis Bryant: Study design, data acquisition (contributing patients), data interpretation, revising manuscript. Approve the final version of the manuscript. Ludvig Muren: Study design, data interpretation, project management, revising manuscript. Approves the final version of the manuscript.

## CONFLICT OF INTEREST STATEMENT

The authors declare no conflicts of interest.

## Supporting information



Supporting Information
