# Supplementary material for: Modelling of a double‐scattering proton therapy nozzle using the FLUKA Monte Carlo code and analysis of linear energy transfer in patients treated for prostate cancer
Source: J Appl Clin Med Phys. 2025 Mar 19;26(6):e70032. doi: 10.1002/acm2.70032 (PMC12148760; doi:10.1002/acm2.70032)
Supplement: Supplementary file 1 — Supporting Information [file ACM2-26-e70032-s001.docx]

# Supplementary material

# Title: Modelling of a double-scattering proton therapy nozzle using the FLUKA Monte Carlo code and analysis of linear energy transfer in patients treated for prostate cancer


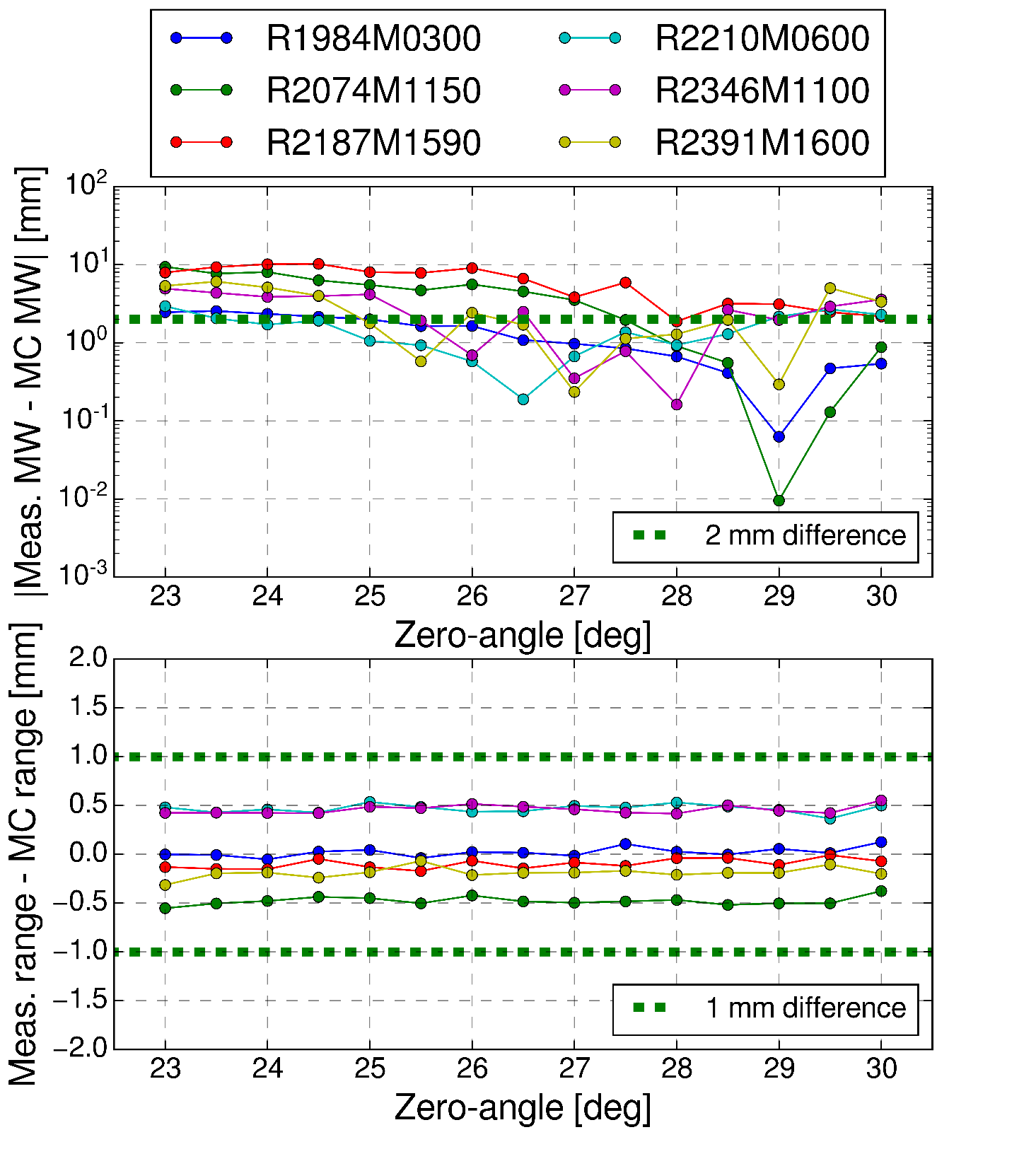


Supplementary figure A: Top-panel: Absolute differences in modulation widths (MW) between FLUKA MC simulations and measurements as a function of MC simulation zero-angle for range-modulation wheel 2. Note the dashed green line indicating a difference of 2 mm. Bottom panel: Range differences between FLUKA MC simulations and measurements as a function of MC simulation zero-angle for range-modulation wheel 2. The difference is calculated as the FLUKA MC range subtracted from the measured range and the dashed green lines indicate plus and minus 1 mm range. The requested ranges and modulation widths are written as RxxxxMyyyy, where xxxx is the range in 10^-1^ mm and yyyy is the modulation width in 10^-1^ mm.


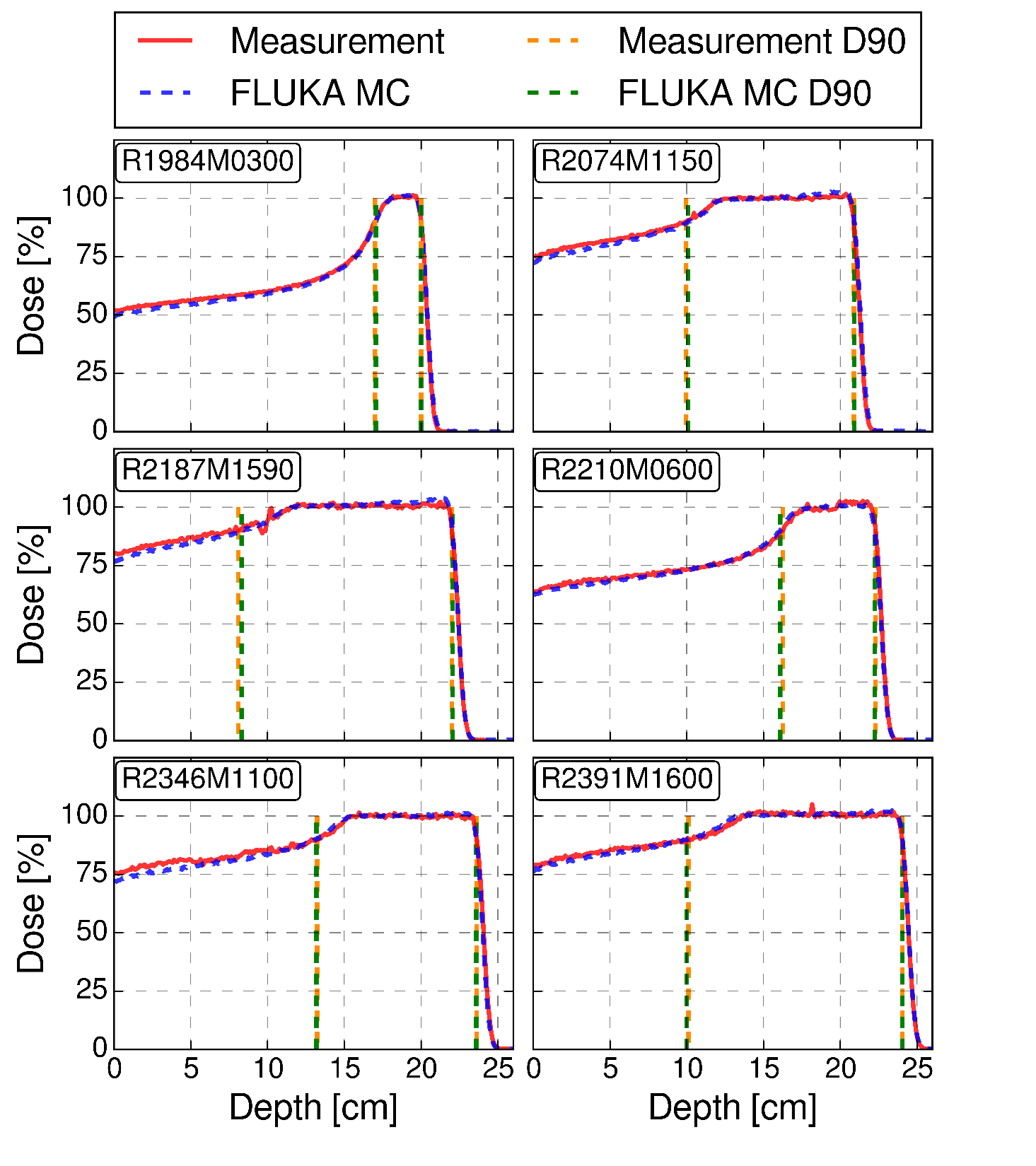
Supplementary figure B: Dose depth curves for all measured and simulated SOBPs using range modulation wheel 2. The dashed blue lines represent the FLUKA MC calculated dose, and the red line represents measurements. The dashed vertical lines represent the D90% ranges (proximal and distal) for FLUKA MC (green) and measurements (orange). The requested ranges and modulation widths are written as RxxxxMyyyy in the upper left corners, where xxxx is the range in 10^-1^ mm and yyyy is the modulation width in 10^-1^ mm.
